# Transparency of Reporting in Molecular Diagnostics

**DOI:** 10.3390/ijms140815878

**Published:** 2013-07-30

**Authors:** Stephen Bustin

**Affiliations:** Postgraduate Medical Institute, Anglia Ruskin University, Chelmsford CM1 1SQ, UK; E-Mail: stephen.bustin@anglia.ac.uk; Tel.: +44-0-845-196-4845

The major advances made over the past few years in molecular and cell biology are providing a progressively more detailed understanding of the molecular pathways that control normal processes and become dysregulated in disease [1]. This has resulted in the documentation of numerous genetic, epigenetic, transcriptomic, proteomic and metabolomic biomarkers that promise earlier disease detection, more accurate patient stratification and better prognosis [2–5]. Furthermore, molecular fingerprinting of diseases can be predictive of drug response and so assist with specific targeting of drugs against disease-associated molecules and function [6].

This, together with the continuous appearance of new technologies, is leading to the introduction of new tools and emergent applications for molecular diagnostics (MDx), usually defined as the detection of changes associated with certain states of health or disease using molecular methods [7]. MDx has the potential to transform modern health care by combining speed and accuracy with low cost to improve health outcomes for individual patients as well as reducing expenditure by health care providers. However, it is also important to consider the implications of the often-unexpected complexity and heterogeneity of diseases that have become apparent as a result of their molecular dissections [2,8].

MDx has become more than just analysis of genetic content for disease information, and makes use of a range of techniques including isothermal amplification methods [9], nucleic acid sequencing [10], high resolution melt analysis [11], DNA microarrays [12], tissue arrays [13], mass spectrometry [14], nanoparticles [15] and fluorescence *in situ* hybridisation [16]. Perhaps the most ubiquitous molecular technique is the real-time quantitative polymerase chain reaction (qPCR) [17], which can detect DNA, RNA (reverse transcription (RT)-qPCR) and proteins (proximity ligation/extension assay). Together, these methods address three distinct categories of clinical questions:

- Pathogen detection, for example by screening for pathogen-specific nucleic acids, proteins or metabolites. One of the strengths of molecular diagnostic techniques is their capacity to vary their specificity from the detection of a single strain (e.g., *Escherichia coli* O157:H7) to that of a species (e.g., *Aspergillus*).
- Expression profiling, which aims to detect disease-associated changes to coding and non-coding cellular RNA and which is beginning to include the analysis of protein-based markers [18].
- Genetic testing, which generally refers to screening for host-derived, epigenetically or genetically altered DNA [19].

These questions are addressed in the context of many clinical application areas. Arguably at the forefront are infectious diseases, where MDx has become part of the routine workload in most clinical laboratories [20] and is having a major impact on clinical decision-making [21]. Sepsis, for example, is among the most common causes of death in hospitals and MDx promises to transform sepsis from a physiologic syndrome into a group of distinct biochemical disorders, thus helping in the development of better diagnostic tools and effective adjunctive sepsis therapies [22]. Several molecular detection techniques are in use for the reliable identification of a targeted pathogen, with qPCR today’s “gold” standard of diagnosis [23].

Oncological applications are also on the increase [24], based on the discovery of differential mRNA and miRNA expression patterns, cell surface proteins and other molecular attributes associated with cancers. They are closely linked to pharmacogenomics uses [25], which aim to correlate the influence of genetic variation with drug response and so develop drugs that target molecular pathways implicated in diseased cells without affecting normal cells. These novel tests may help realise the most anticipated development from MDx: personalised medicine, which optimises patient outcomes and healthcare use by utilising an individual’s genetic makeup to tailor an individual treatment plan that is administered at the right time and at the right dose. Examples include breast cancers that overexpress HER2 [26], colorectal cancers that are mutated in the BRAF gene [27], chronic myeloid leukaemias that do not respond to the tyrosine kinase inhibitor imatinib [28] and gastrointestinal stromal tumours with selective c-kit oncogene activating mutations [29].

Third, MDx are becoming of increasing importance for the screening of genetic alterations [30], an application whose importance can only increase and is certain to influence the management of diseases [31].

The IJMS has developed a reputation for the quality of its contributions and proposes to develop an enhanced focus on this rapidly expanding area of molecular science. We encourage the submission of high quality reviews, research articles and short communications that place special emphasis on the translation of research discoveries into practice, describe novel or improved technologies and their applications, and are characterised by an emphasis on transparency of reporting. This includes manuscripts discussing new bioinformatic concepts and programs that advance our understanding of the link between molecular data and physiology or pathophysiology.

A major problem with many peer-reviewed publications is that they propagate conclusions that cannot be reproduced elsewhere, and so contribute to the challenge of distinguishing biologically valid conclusions from those that are based on technical errors, analytical inaccuracies or interpretative misinterpretations [32]. An important reason for this is the reluctance of many journals to provide sufficient space for the publication of detailed experimental protocols, denying readers an opportunity to assess the reliability and validity of the experimental approach and protocol details.

One area of particular concern has been the quantification of nucleic acids by qPCR, whose superficial simplicity, sensitivity and specificity make it the most widely used method for MDx applications. Those attributes are gainsaid by their actual complexity and inconsistency, which in practice means that the biological relevance of many published qPCR-based results is open to question, with reported differences caused by technical, rather than actual, variability [32]. There has been an extensive effort over the last twelve years to make researchers aware of the pitfalls of this technology [33–43], culminating in the publication of guidelines for the minimum information for the publication of qPCR (MIQE) [44] and digital PCR (dPCR, dMIQE) [45] experiments. These guidelines aim to enable authors to design and report qPCR experiments that have greater inherent value, allow reviewers and editors to measure the technical quality of submitted manuscripts against an established yardstick and facilitate easier replication of experiments described in published studies that follow these guidelines. They have become widely accepted, with more than 1600 citations in the peer-reviewed literature, and have paved the way for the recent editorial announcement in Nature and the Nature research journals, which admit their failure to exert sufficient scrutiny over the results that they publish and acknowledge that they have not published enough information for other researchers to assess results properly [46]. There have been editorials in BioMedCentral (BMC) Molecular Biology [47] and the Veterinary Journal [48] promoting the idea of the submission of comprehensive experimental protocols, with Nucleic Acids Research, Peer J, Molecular Medicine, European Urology, The Journal of Clinical Microbiology, The Journal of Molecular Medicine as well as Reproduction and Fertility and Development recommending and Clinical Chemistry requiring adherence to the essential MIQE parameters. Other techniques used in MDx, including microarrays [49] and mass spectrometry [50], suffer from similar challenges that are quite familiar to experts but less obvious to the general reviewer or reader of a publication.

Consequently, the editorial team reviewing and accepting publications in the IJMS will implement measures that safeguard the transparency and quality of any publication appearing in this journal. This means that there will be emphasis on the publication of comprehensive technical detail sufficient to allow the interested reader to reproduce accurately any experimental protocols. For nucleic acid-based techniques, there will be particular emphasis on quality assessment—critical especially for methods targeting RNA [51]—assay design and efficiency and normalisation procedures. Ideally, this information will be published in the form of a supplementary file that will include the following information:

- Nucleic acid purity: absence of inhibitors that can interfere with the reverse transcription, PCR or hybridisation stages of assays. This is easily established by using a nucleic acid spike, e.g., the SPUD assay [52], or a 10-fold dilution test to check for inhibition.
- RNA integrity: established by means of a microfluidic analysis of the RNA [53] or a 3′:5′ assessment of target or reference RNAs [54].
- Assay design/PCR efficiency: primers and probes used for PCR reactions should be published with individual papers and not just be referenced, since the referenced publication is frequently not on open access, making it more difficult than necessary to identify the details of the assays used. It is also essential to publish information about the efficiency of the PCR assay, as this provides useful information about the robustness and sensitivity of the assay in question.
- Normalisation: this should be against validated reference genes [55] appropriate for the fold-differential expression claimed for mRNAs [56] as well as for miRNAs [57,58].

These criteria are especially aimed at publications utilising RT-qPCR and qPCR-based results, since they form the vast majority of all reported MDx data. However, there is an obvious need to provide protocols of similar transparency and completeness for other techniques; for example, evidence should be provided for absence of amplification bias when pre-amplifying limited sample material, and evidence of appropriate verification is required for the new high-throughput RNA Seq or small-RNA Seq data. For microarray data there will need to be some indication of the accuracy, precision and specificity of the data presented for publication [59], ideally with corroboration by an alternative method such as RT-qPCR. For mass spectrometry there will need to be evidence for satisfactory peak shape, mass resolution and calibration as well as a noise-free background spectrum.

All of the information requested will become available during the course of an appropriately conducted experiment; hence it does not constitute an additional workload. Sharing those basic data will ensure that the journal publishes results and conclusions whose underlying technical fitness is beyond doubt, allowing readers to focus on the biological and clinical relevance of the results. Ultimately, this approach will enhance the reputation of both journals and publications, which is in all our interest.
